# 4-Hydroxy­benzohydrazide

**DOI:** 10.1107/S1600536809025094

**Published:** 2009-07-04

**Authors:** Rifat Ara Jamal, Uzma Ashiq, Muhammad Nadeem Arshad, Zahida Tasneem Maqsood, Islam Ullah Khan

**Affiliations:** a Department of Chemistry, University of Karachi, Karachi 75270, Pakistan; b Department of Chemistry, Government College University, Lahore, Pakistan

## Abstract

In the title compound, C_7_H_8_N_2_O_2_, the mean planes of the benzene ring and the planar hydrazide group are inclined at 25.75 (6)° with respect to each other. The structure is stabilized by inter­molecular N—H⋯O and O—H⋯N hydrogen bonds.

## Related literature

For related structures see: Ashiq, Jamal *et al.* (2008 ▶, 2009 ▶); Hanif *et al.* (2007 ▶); Jamal *et al.* (2008 ▶); Kallel *et al.* (1992 ▶); Saraogi *et al.* (2002 ▶). For the biological activity of hydrazides, see: Ara *et al.* (2007 ▶); Ashiq, Ara *et al.* (2008 ▶); Maqsood *et al.* (2006 ▶).
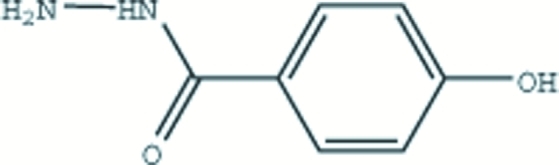


## Experimental

### 

#### Crystal data

C_7_H_8_N_2_O_2_
*M* *_r_* = 152.15
Monoclinic, 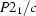

*a* = 5.0587 (2) Å
*b* = 17.2149 (9) Å
*c* = 7.8178 (5) Å
β = 93.489 (2)°
*V* = 679.55 (6) Å^3^
*Z* = 4
Mo *K*α radiation
μ = 0.11 mm^−1^
*T* = 296 K
0.32 × 0.18 × 0.12 mm

#### Data collection

Bruker Kappa APEXII CCD diffractometer
Absorption correction: multi-scan (*SADABS*; Bruker, 2005 ▶) *T*_min_ = 0.965, *T*_max_ = 0.992
7324 measured reflections
1697 independent reflections
1348 reflections with *I* > 2σ(*I*)
*R*_int_ = 0.023

#### Refinement

*R*[*F* ^2^ > 2σ(*F* ^2^)] = 0.039
*wR*(*F* ^2^) = 0.118
*S* = 1.06
1697 reflections
107 parameters
H atoms treated by a mixture of independent and constrained refinement
Δρ_max_ = 0.36 e Å^−3^
Δρ_min_ = −0.20 e Å^−3^

### 

Data collection: *APEX2* (Bruker, 2007 ▶); cell refinement: *SAINT* (Bruker, 2007 ▶); data reduction: *SAINT*; program(s) used to solve structure: *SHELXS97* (Sheldrick, 2008 ▶); program(s) used to refine structure: *SHELXL97* (Sheldrick, 2008 ▶); molecular graphics: *ORTEP-3 for Windows* (Farrugia, 1997 ▶); software used to prepare material for publication: *SHELXL97*.

## Supplementary Material

Crystal structure: contains datablocks I, global. DOI: 10.1107/S1600536809025094/pv2171sup1.cif

Structure factors: contains datablocks I. DOI: 10.1107/S1600536809025094/pv2171Isup2.hkl

Additional supplementary materials:  crystallographic information; 3D view; checkCIF report

## Figures and Tables

**Table 1:** Hydrogen-bond geometry (Å, °)

*D*—H⋯*A*	*D*—H	H⋯*A*	*D*⋯*A*	*D*—H⋯*A*
N1—H1⋯O2^i^	0.86	2.13	2.9243 (14)	153
O1—H1*A*⋯N2^ii^	0.82	1.98	2.7852 (16)	174
N2—H12⋯O1^iii^	0.89 (2)	2.37 (2)	3.223 (2)	160
N2—H22⋯O2^iv^	0.90 (2)	2.22 (2)	3.056 (2)	155

Symmetry codes: (i) 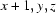; (ii) 
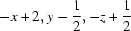
; (iii) 
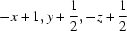
; (iv) 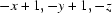.

**Fig. 1.**
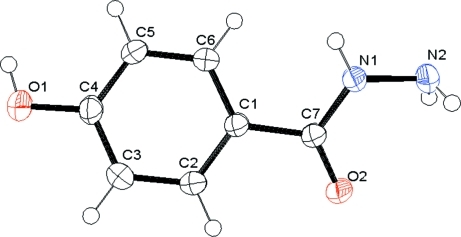
ORTEP plot of the title compound with the ellipsoids drawn at the 50% probability level.

**Fig. 2.**
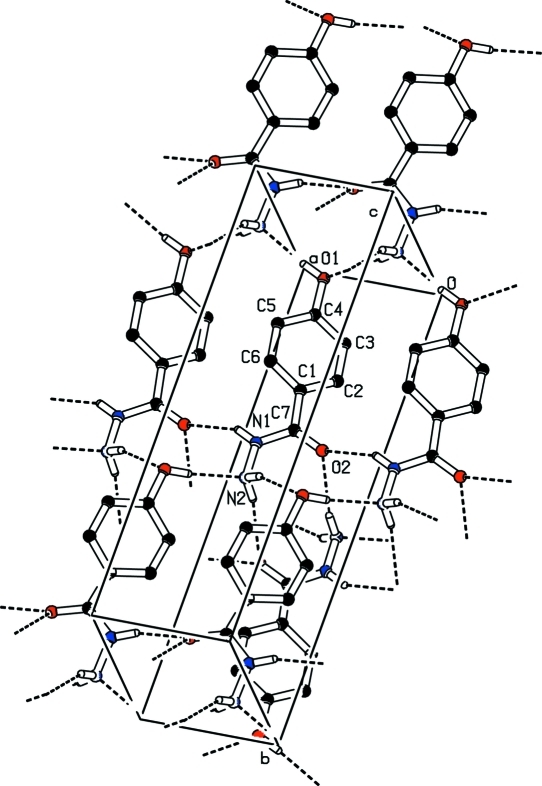
A packing diagram of (I). Hydrogen bonds are shown by dashed lines.

## supplementary crystallographic information

### Comment

Hydrazides are known to have different biological activities (Ashiq, Ara *et al.*, 2008; Ara *et al.*, 2007). In order to study the biological activity of 4-hydroxybenzohydrazide, we undertook the synthesis of the title compound, (I), and report its crystal structure in this paper.  The title compound was found to be antifungal (Maqsood *et al.*, 2006). The crystal structures of benzhydrazide (Kallel *et al.*, 1992), *para*-chloro (Saraogi *et al.*, 2002), *para*-bromo (Ashiq, Jamal *et al.*, 2008), *para*-iodo (Jamal *et al.*, 2008) and *para*-methoxy (Ashiq, Jamal *et al.*, 2009) analogues of (I) have already been reported. The structure of (I) is isomorphous with its 3-hydroxy analogue (Hanif *et al.*, 2007).

The molecular structure of (I) has been presented in Fig. 1. The bond distances and bond angles in (I) are similar to the corresponding distances and angles reported in the structures quoted above. In (I), the mean-planes of the benzene ring (C1–C6) and planar hydrazide group (N1/N2/O2/C7) are inclined at 25.75 (6)° with respect to each other. The molecular packing diagram (Fig. 2) shows the presence of four intermolecular hydrogen bonds of the type N—H···O and O—H···N (details are given in Table 1).

### Experimental

All reagent-grade chemicals were obtained from Aldrich and Sigma Chemical companies and were used without further purification. To a solution of ethyl-4-hydroxybenzoate (3.32 g, 20 mmol) in 75 ml e thanol, hydrazine hydrate (5.0 ml, 100 mmol) was added. The mixture was refluxed for 5 h and a solid was obtained upon removal of the solvent by rotary evaporation. The resulting solid was washed with hexane to afford 4-hydroxybenzohydrazide (yield 65%) (Maqsood *et al.*, 2006).

### Refinement

H atoms were positioned geometrically, with aromatic C—H, O—H and N1—H1 distances 0.93, 0.82 and  0.86 Å, respectively, and constrained to ride on their parent atoms. The H-atoms attached to N2 atom were taken from Fourier synthesis and their coordinates were refined. The thermal parameter of H-atoms of was taken 1.2 times the equivalent isotropic displacement parameters of their parent C and N-atoms and 1.5 times the O-atom.

### Crystal data

**Table:**

C_7_H_8_N_2_O_2_	*F*(000) = 320
*M**_r_* = 152.15	*D*_x_ = 1.487 Mg m^−^^3^
Monoclinic, *P*2_1_/*c*	Mo *K*α radiation, λ = 0.71073 Å
Hall symbol: -P 2ybc	Cell parameters from 2993 reflections
*a* = 5.0587 (2) Å	θ = 2.9–28.3°
*b* = 17.2149 (9) Å	µ = 0.11 mm^−^^1^
*c* = 7.8178 (5) Å	*T* = 296 K
β = 93.489 (2)°	Needle, colourless
*V* = 679.55 (6) Å^3^	0.32 × 0.18 × 0.12 mm
*Z* = 4	

### Data collection

**Table:**

Bruker Kappa APEX2 CCD diffractometer	1697 independent reflections
Radiation source: fine-focus sealed tube	1348 reflections with *I* > 2σ(*I*)
graphite	*R*_int_ = 0.023
ω scans	θ_max_ = 28.3°, θ_min_ = 2.4°
Absorption correction: multi-scan (*SADABS*; Bruker, 2005)	*h* = −6→6
*T*_min_ = 0.965, *T*_max_ = 0.992	*k* = −22→23
7324 measured reflections	*l* = −10→9

### Refinement

**Table:**

Refinement on *F*^2^	Primary atom site location: structure-invariant direct methods
Least-squares matrix: full	Secondary atom site location: difference Fourier map
*R*[*F*^2^ > 2σ(*F*^2^)] = 0.039	Hydrogen site location: inferred from neighbouring sites
*wR*(*F*^2^) = 0.118	H atoms treated by a mixture of independent and constrained refinement
*S* = 1.06	*w* = 1/[σ^2^(*F*_o_^2^) + (0.0613*P*)^2^ + 0.1539*P*] where *P* = (*F*_o_^2^ + 2*F*_c_^2^)/3
1697 reflections	(Δ/σ)_max_ < 0.001
107 parameters	Δρ_max_ = 0.36 e Å^−^^3^
0 restraints	Δρ_min_ = −0.20 e Å^−^^3^

### Special details

**Table:**

Geometry. All e.s.d.'s (except the e.s.d. in the dihedral angle between two l.s. planes) are estimated using the full covariance matrix. The cell e.s.d.'s are taken into account individually in the estimation of e.s.d.'s in distances, angles and torsion angles; correlations between e.s.d.'s in cell parameters are only used when they are defined by crystal symmetry. An approximate (isotropic) treatment of cell e.s.d.'s is used for estimating e.s.d.'s involving l.s. planes.
Refinement. Refinement of *F*^2^ against ALL reflections. The weighted *R*-factor *wR* and goodness of fit *S* are based on *F*^2^, conventional *R*-factors *R* are based on *F*, with *F* set to zero for negative *F*^2^. The threshold expression of *F*^2^ > σ(*F*^2^) is used only for calculating *R*-factors(gt) *etc*. and is not relevant to the choice of reflections for refinement. *R*-factors based on *F*^2^ are statistically about twice as large as those based on *F*, and *R*- factors based on ALL data will be even larger.

### Fractional atomic coordinates and isotropic or equivalent isotropic displacement parameters (Å^2^)

**Table:**

	*x*	*y*	*z*	*U*_iso_*/*U*_eq_	
O1	0.7565 (2)	0.04052 (5)	0.14740 (17)	0.0478 (3)	
H1A	0.8946	0.0291	0.2020	0.072*	
O2	0.36833 (17)	0.38981 (5)	0.10180 (14)	0.0364 (3)	
N1	0.8045 (2)	0.40995 (6)	0.15266 (16)	0.0331 (3)	
H1	0.9606	0.3900	0.1554	0.040*	
N2	0.7783 (2)	0.49074 (6)	0.1772 (2)	0.0381 (3)	
H12	0.647 (4)	0.4969 (10)	0.247 (2)	0.046*	
H22	0.736 (3)	0.5124 (10)	0.075 (2)	0.046*	
C1	0.6499 (2)	0.27877 (7)	0.12927 (16)	0.0244 (3)	
C2	0.4705 (2)	0.22850 (7)	0.04589 (17)	0.0299 (3)	
H2	0.3217	0.2486	−0.0145	0.036*	
C3	0.5095 (3)	0.14912 (8)	0.05121 (18)	0.0335 (3)	
H3	0.3892	0.1161	−0.0066	0.040*	
C4	0.7289 (3)	0.11891 (7)	0.14320 (18)	0.0304 (3)	
C5	0.9081 (2)	0.16847 (7)	0.22869 (19)	0.0325 (3)	
H5	1.0545	0.1484	0.2915	0.039*	
C6	0.8685 (2)	0.24751 (7)	0.22038 (18)	0.0307 (3)	
H6	0.9904	0.2805	0.2768	0.037*	
C7	0.5947 (2)	0.36334 (7)	0.12527 (16)	0.0255 (3)	

### Atomic displacement parameters (Å^2^)

**Table:**

	*U*^11^	*U*^22^	*U*^33^	*U*^12^	*U*^13^	*U*^23^
O1	0.0407 (6)	0.0216 (5)	0.0792 (9)	0.0012 (4)	−0.0124 (5)	0.0027 (5)
O2	0.0201 (4)	0.0278 (5)	0.0605 (7)	0.0024 (3)	−0.0032 (4)	0.0034 (4)
N1	0.0203 (5)	0.0213 (5)	0.0574 (7)	0.0009 (4)	−0.0008 (5)	−0.0007 (5)
N2	0.0305 (6)	0.0206 (5)	0.0627 (9)	−0.0015 (4)	−0.0020 (6)	0.0009 (5)
C1	0.0202 (5)	0.0231 (5)	0.0299 (6)	0.0007 (4)	0.0011 (4)	0.0016 (5)
C2	0.0238 (6)	0.0277 (6)	0.0372 (7)	−0.0002 (4)	−0.0061 (5)	0.0024 (5)
C3	0.0299 (6)	0.0277 (6)	0.0418 (8)	−0.0046 (5)	−0.0057 (5)	−0.0017 (5)
C4	0.0288 (6)	0.0208 (6)	0.0418 (7)	−0.0002 (5)	0.0037 (5)	0.0024 (5)
C5	0.0240 (6)	0.0274 (6)	0.0450 (8)	0.0033 (5)	−0.0061 (5)	0.0045 (5)
C6	0.0238 (6)	0.0266 (6)	0.0408 (7)	−0.0009 (5)	−0.0067 (5)	−0.0006 (5)
C7	0.0210 (5)	0.0241 (6)	0.0311 (6)	0.0011 (4)	0.0004 (4)	0.0013 (5)

### Geometric parameters (Å, °)

**Table:**

O1—C4	1.3569 (14)	C1—C7	1.4824 (16)
O1—H1A	0.8200	C2—C3	1.3809 (17)
O2—C7	1.2356 (14)	C2—H2	0.9300
N1—C7	1.3376 (15)	C3—C4	1.3866 (18)
N1—N2	1.4113 (15)	C3—H3	0.9300
N1—H1	0.8600	C4—C5	1.3864 (18)
N2—H12	0.89 (2)	C5—C6	1.3762 (17)
N2—H22	0.90 (2)	C5—H5	0.9300
C1—C6	1.3870 (17)	C6—H6	0.9300
C1—C2	1.3876 (17)		
			
C4—O1—H1A	109.5	C2—C3—H3	120.2
C7—N1—N2	122.18 (10)	C4—C3—H3	120.2
C7—N1—H1	118.9	O1—C4—C5	122.50 (12)
N2—N1—H1	118.9	O1—C4—C3	117.60 (12)
N1—N2—H12	106.3 (11)	C5—C4—C3	119.90 (12)
N1—N2—H22	108.1 (11)	C6—C5—C4	119.78 (12)
H12—N2—H22	110.4 (17)	C6—C5—H5	120.1
C6—C1—C2	118.52 (11)	C4—C5—H5	120.1
C6—C1—C7	122.35 (11)	C5—C6—C1	121.13 (12)
C2—C1—C7	119.05 (11)	C5—C6—H6	119.4
C3—C2—C1	120.98 (12)	C1—C6—H6	119.4
C3—C2—H2	119.5	O2—C7—N1	121.45 (11)
C1—C2—H2	119.5	O2—C7—C1	122.48 (11)
C2—C3—C4	119.68 (12)	N1—C7—C1	116.04 (10)
			
C6—C1—C2—C3	−0.8 (2)	C2—C1—C6—C5	0.0 (2)
C7—C1—C2—C3	−177.79 (12)	C7—C1—C6—C5	176.81 (12)
C1—C2—C3—C4	1.0 (2)	N2—N1—C7—O2	7.9 (2)
C2—C3—C4—O1	178.80 (12)	N2—N1—C7—C1	−170.63 (12)
C2—C3—C4—C5	−0.2 (2)	C6—C1—C7—O2	−152.94 (13)
O1—C4—C5—C6	−179.61 (13)	C2—C1—C7—O2	23.90 (18)
C3—C4—C5—C6	−0.6 (2)	C6—C1—C7—N1	25.57 (18)
C4—C5—C6—C1	0.8 (2)	C2—C1—C7—N1	−157.60 (12)

### Hydrogen-bond geometry (Å, °)

**Table:**

*D*—H···*A*	*D*—H	H···*A*	*D*···*A*	*D*—H···*A*
N1—H1···O2^i^	0.86	2.13	2.9243 (14)	153
O1—H1A···N2^ii^	0.82	1.98	2.7852 (16)	174
N2—H12···O1^iii^	0.89 (2)	2.37 (2)	3.223 (2)	160
N2—H22···O2^iv^	0.90 (2)	2.22 (2)	3.056 (2)	155

Symmetry codes: (i) *x*+1, *y*, *z*; (ii) −*x*+2, *y*−1/2, −*z*+1/2; (iii) −*x*+1, *y*+1/2, −*z*+1/2; (iv) −*x*+1, −*y*+1, −*z*.
